# Type I interferon pathway in pediatric systemic lupus erythematosus

**DOI:** 10.1007/s12519-024-00811-4

**Published:** 2024-06-25

**Authors:** Yu Zhou, Hong-Mei Song

**Affiliations:** 1 Department of Pediatrics, Peking Union Medical College Hospital, Chinese Academy of Medical Sciences, Beijing, China; 2 State Key Laboratory of Complex Severe and Rare Diseases, Peking Union Medical College Hospital, Beijing, China

**Keywords:** Interferonopathies, Pediatric systemic lupus erythematosus, Type I interferon

## Abstract

**Background:**

The role of type I interferon (IFN-I) signaling in systemic lupus erythematosus (SLE) has been well established. However, unanswered questions remain regarding the applicability of these findings to pediatric-onset SLE. The aim of this review is to provide an overview of the novel discoveries on IFN-I signaling in pediatric-onset SLE.

**Data sources:**

A literature search was conducted in the PubMed database using the following keywords: “pediatric systemic lupus erythematosus” and “type I interferon”.

**Results:**

IFN-I signaling is increased in pediatric SLE, largely due to the presence of plasmacytoid dendritic cells and pathways such as cyclic GMP-AMP synthase–stimulator of interferon genes–TANK-binding kinase 1 and Toll-like receptor (TLR)4/TLR9. Neutrophil extracellular traps and oxidative DNA damage further stimulate IFN-I production. Genetic variants in IFN-I-related genes, such as IFN-regulatory factor 5 and tyrosine kinase 2, are linked to SLE susceptibility in pediatric patients. In addition, type I interferonopathies, characterized by sustained IFN-I activation, can mimic SLE symptoms and are thus important to distinguish. Studies on interferonopathies also contribute to exploring the pathogenesis of SLE. Measuring IFN-I activation is crucial for SLE diagnosis and stratification. Both IFN-stimulated gene expression and serum IFN-α2 levels are common indicators. Flow cytometry markers such as CD169 and galectin-9 are promising alternatives. Anti-IFN therapies, such as sifalimumab and anifrolumab, show promise in adult patients with SLE, but their efficacy in pediatric patients requires further investigation. Janus kinase inhibitors are another treatment option for severe pediatric SLE patients.

**Conclusions:**

This review presents an overview of the IFN-I pathway in pediatric SLE. Understanding the intricate relationship between IFN-I and pediatric SLE may help to identify potential diagnostic markers and targeted therapies, paving the way for improved patient care and outcomes.

**Graphical Abstract:**

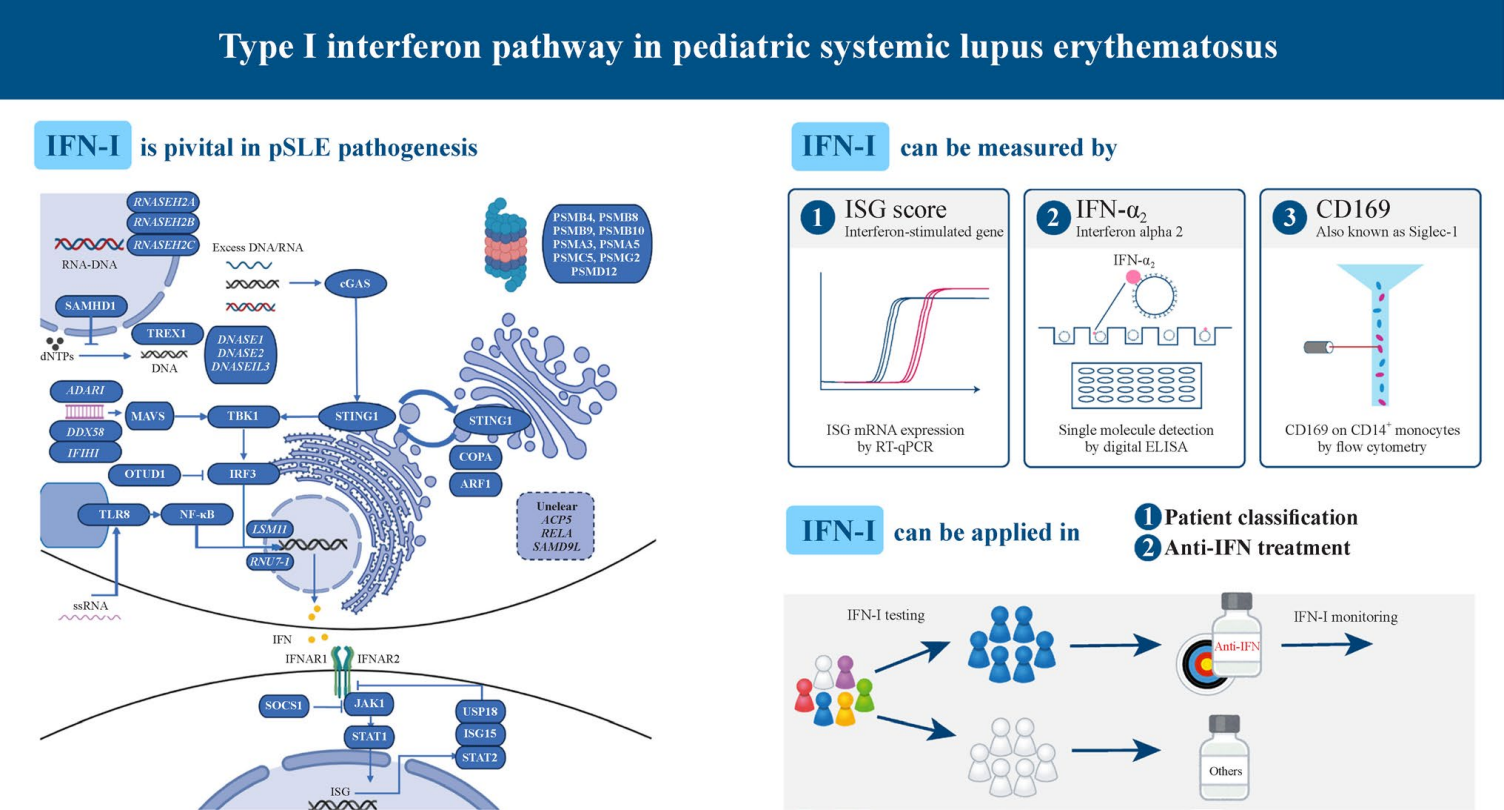

## Introduction

Interferons (IFNs) constitute a crucial class of cytokines with significant roles in the immune response. They can be categorized into three families based on their structural characteristics, immunomodulatory functions, and the cells responsible for their secretion. Type I interferon (IFN-I) represents one of these subfamilies and include 17 proteins: 13 for IFN-α, IFN-β, IFN-ω, IFN-ε, and IFN-κ [1]. Generally, the levels of IFN-I peak during the initial days following acute viral infections and return to normal once the virus has been cleared, indicating a transient and time-limited response [2]. However, elevated IFN-I was observed in systemic lupus erythematosus (SLE) patients [3]. Over the past few decades, research has shed light on the role of IFN-I in the context of SLE [4]. It has been well established that IFN-I signaling is activated in SLE, with elevated levels of IFN-I contributing to the progression of the disease through various mechanisms. These mechanisms include the induction of autoreactive T cells, the promotion of autoantibody production, and the upregulation of proinflammatory cytokine expression [5]. In addition, anomalies in other pathways in SLE, such as complement deficiency, could also cause secondary overexpression of IFN-I [6]. However, the majority of these studies have been conducted in adult populations, leaving uncertainties regarding whether similar conditions apply to pediatric-onset SLE. Therefore, this review aimed to explore the relationship between the IFN-I pathway and SLE, focusing specifically on investigations conducted in pediatric patients.

## Type I interferon signaling in classic systemic lupus erythematosus

Multiple studies have established a robust association between elevated IFN-I levels and SLE. Aberrant IFN-I signaling is thought to contribute to the perpetuation of autoimmunity in SLE patients [7]. Several mechanisms underlying IFN-I dysregulation in SLE include increased IFN-I production by plasmacytoid dendritic cells, defective IFN-I receptor signaling, and a failure to negatively regulate IFN-I production [8]. Notable genes involved in IFN-I signaling, such as IFN-regulatory factors (IRFs) [9] and signal transducer and activator of transcription (STATs) [10], have been linked to SLE susceptibility. IFN-I plays a multifaceted role in SLE pathogenesis. It promotes B-cell activation, disrupts immune tolerance, and drives the differentiation of autoreactive T cells [11–13]. Additionally, IFN-I induces the expression of various proinflammatory cytokines, amplifying the inflammatory cascade in affected tissues [14]. These collective effects contribute to the production of autoantibodies and immune complex deposition, which are central to the immunopathology of SLE.

In pediatric SLE, the overproduction of IFN-I is also prominent. Most IFN-I is produced by plasmacytoid dendritic cells and usually depends on the cyclic GMP-AMP synthase–stimulator of interferon genes–TANK-binding kinase 1 (cGAS-STING-TBK1) pathway as well as the Toll-like receptor (TLR)4/TLR9 pathways [15]. The former represents an instinct nucleic acid-sensing pathway, while the latter is usually activated during bacterial infections [16, 17]. A recent study revealed that in pediatric SLE, the presence of undigested nucleic acids and bacterial stimulation can potentially collaborate in a positive feedback loop, ultimately leading to dysregulated IFN production [18].

The triggers of IFN-I overproduction in SLE could vary, and neutrophil extracellular traps (NETs) might be important. A study demonstrated that mature neutrophils from pediatric SLE patients were primed in vivo by IFN-I and underwent cell death when exposed to SLE-derived anti-ribonucleoprotein antibodies. This resulted in the release of NETs. Then, NETs activate plasmacytoid dendritic cells to enhance the uptake and recognition of mammalian DNA, prompting them to produce elevated levels of IFN-α [19]. Another possible trigger of IFN-I production is oxidative DNA damage. Experiments revealed that defects in *OGG1*, a DNA repair enzyme that repairs 8-OH-dG DNA lesions, which accumulates in oxidized DNA [20], enhanced IFN-driven gene expression and was associated with increased autoantibodies. Furthermore, the expression of *OGG1* was notably lower in lesional skin than in non-lesional skin in patients diagnosed with discoid lupus [21].

In pediatric SLE, the overproduction of IFN-I could also be a result of impaired inhibition of related pathways. An Australian group elucidated the functional implications of these rare and infrequent missense variants in the interacting proteins B-lymphoid kinase and B-cell scaffold protein with ankyrin repeats 1, which were found either individually or in combination in a significant proportion of individuals with lupus. The rare variants identified in SLE patients, as opposed to those exclusively found in the control group, hinder the suppression of IRF5 and IFN in human B-cell lines and augment the presence of pathogenic lymphocytes in mice prone to lupus [22]. In addition, another group found de novo protein kinase C and casein kinase substrate in neurons 1 (PACSIN1) missense variants in a child with SLE [23]. Their study established that PACSIN1 formed a trimolecular complex involving tumor necrosis factor receptor-associated factor 4 (TRAF4) and TRAF6, which plays a crucial role in the regulation of IFN-I. The Q59K mutation in PACSIN1 reduced its interaction with TRAF4. Consequently, this alteration resulted in uncontrolled TRAF6-mediated activation of IFN-I.

In pediatric SLE patients, several variants of IFN-I-related genes were found to be associated with SLE susceptibility, similar to the findings in the adult population. *IRF5* is one such gene that has been identified as an autoimmune susceptibility gene [24]. To date, at least four variants of *IRF5* have been associated with SLE risk [25–29]. Li et al. demonstrated that individuals carrying the *IRF5*-SLE risk haplotype had increased IFN pathway enrichment and decreased reactive oxygen species pathway expression and thus had increased circulating plasmacytoid dendritic cells and plasma cells, as well as elevated spontaneous NETosis [30]. In a recent Mexican study, two tyrosine kinase 2 (TYK2) variants related to infection risk were shown to be protective against SLE [31].

## Type I interferonopathies in monogenic systemic lupus erythematosus

**Fig. 1:**
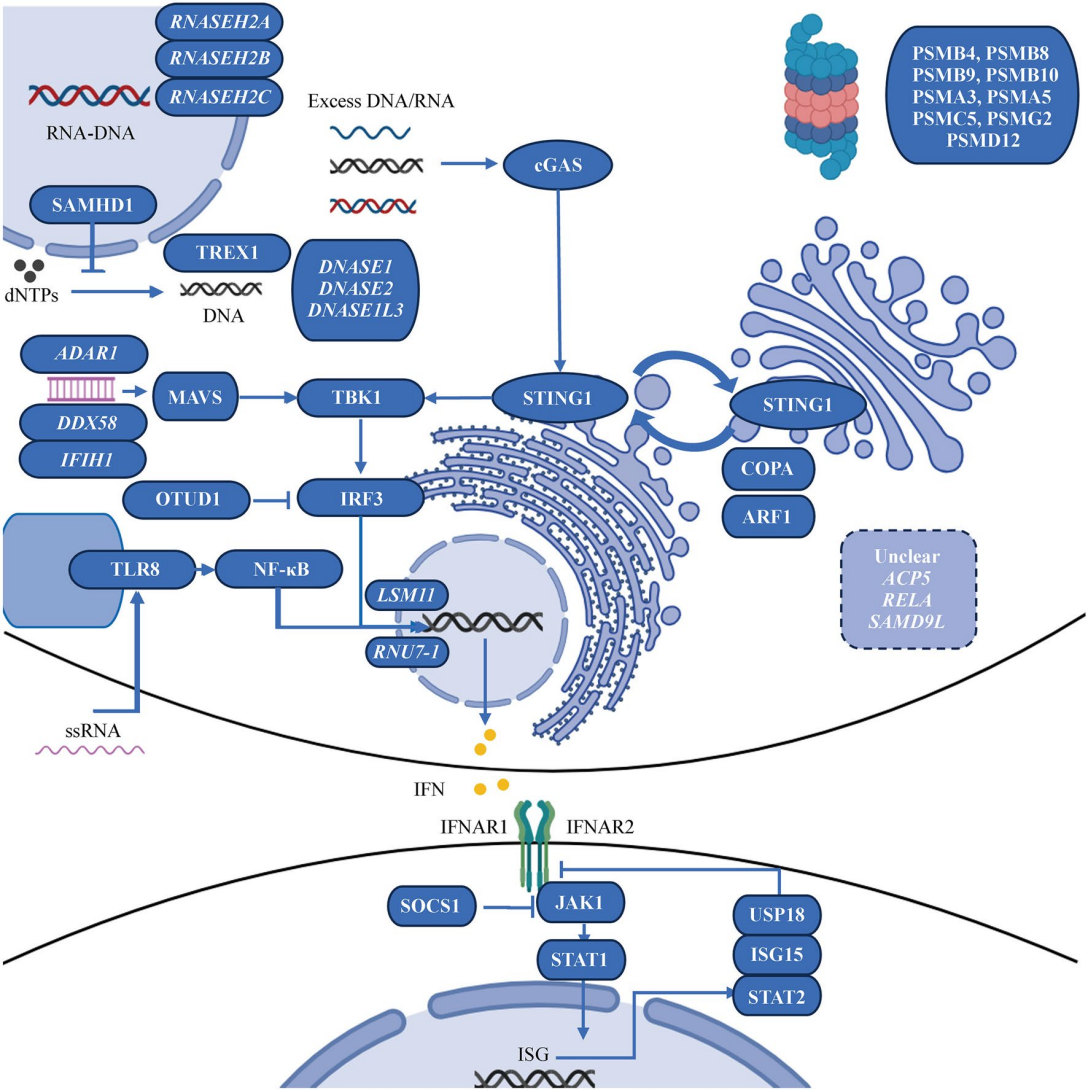
IFN-I pathway and known genes related to type I interferonopathies and monogenic SLE. Ribonuclease H2 (encoded by *RNASEH2A*, *RNASEH2B* and *RNASEH2C*), SAMHD1, TREX1 and DNases (encoded by *DNASE1*, *DNASE2* and *DNASE1L3*, etc.) participate in RNA‒DNA hybrids, deoxynucleotide triphosphates (dNTPs) and single-stranded DNA as well as double-stranded DNA metabolism. Mutations in these genes cause excess accumulation of DNA, while mutations in *LSM11* and *RNU7-1* lead to a disorder of histone stoichiometry, resulting in the sensing of nuclear DNA. Together, these factors activate the interferon pathway through cyclic GMP-AMP synthase (cGAS)-stimulator of interferon genes (STING) signaling via the activation of TANK-binding kinase 1 (TBK1)-interferon regulatory factor 3 (IRF3). Mutations in *STING1*, *COPA* and *ARF1* result in prolonged presence of STING in the Golgi apparatus, leading to constitutive activation of IFN-I. Mutations in *IFIH1* (encoding MDA5) and *DDX58* (encoding RIG-I) cause abnormal sensing of exogenous viral double-stranded RNA (dsRNA), and loss-of-function mutations in *ADAR1* lead to the generation of immunogenic dsRNA. These mutations induce interferon production by activating mitochondrial antiviral signaling protein (MAVS)-TBK1-IRF3. OTUD1 acts as a suppressor by deubiquitinating IRF3 and loss-of-function mutations in *OTUD1*, lead to overactivation of IRF3 and thus upregulation of IFN. Moreover, single-stranded RNA (ssRNA) from viruses and bacteria, detected by TLR8, can trigger the IFN-I response via NF-κB activation. After IFN-I binds to its receptor, JAK1 and TYK2 are phosphorylated and subsequently activate STAT1 to induce the expression of interferon-stimulated genes (ISGs). Gain-of-function mutations in *JAK1* and *STAT1* therefore result in overactivation of IFN-I. USP18, ISG15 and STAT2 participate in the regulation of the IFN pathway by inhibiting signaling downstream of IFNARs, while deficiency of USP18 and ISG15, as well as separation-of-function mutations in *STAT2,* promote the induction of interferon signaling. Currently, there are at least 9 genes related to the proteasome that causes interferonopathies, but the exact underlying mechanism remains unclear. In addition, the relationships of mutations in *ACP5*, *RELA*, and *SAMD9L* with disease remain unknown. *IFN-I* type I interferon, *SLE* systemic lupus erythematosus, *RNASEH2A* ribonuclease H2 subunit A, *SAMHD1* SAM-domain- and HD-domain-containing protein 1, *TREX1* 3′ repair exonuclease 1, *DNASE1L3* deoxyribonuclease 1 like 3, *COPA* coatomer protein complex subunit alpha, *ARF1* ADP-ribosylation factor 1, *IFIH1* interferon induced with helicase C domain 1, *RIG-I* retinoic acid-inducible I, *ADAR1* adenosine deaminase RNA specific 1, *OTUD1* ovarian tumor deubiquitinase 1, *TLR* Toll-like receptor, *NF-κB* nuclear factor-κB, *JAK1* Janus kinase 1, *TYK2* tyrosine kinase 2, *STAT1* signal transducer and activator of transcription 1, *USP18* ubiquitin-specific peptidase 18, *IFNARs* IFN-α receptors, *SOCS1* suppressor of cytokine signaling 1, *SAMD9L* sterile alpha motif domain-containing 9 like, *PSM* proteasome

Monogenic SLE represents a distinct subset of pediatric SLE patients, and the relationship between monogenic SLE and type I interferonopathies is intricate, showing overlapping features that blur the lines between these distinct entities. Monogenic SLE refers to a subset of SLE cases driven primarily by single-gene mutations, often affecting key components of immune pathways, while type I interferonopathies encompass a group of inborn errors of immunity characterized by sustained activation of the IFN-I signaling pathway (Fig. 1). Notably, a considerable number of type I interferonopathies, including Aicardi-Goutières syndrome (AGS), proteasome-associated autoinflammatory syndrome (PRAAS) and STING-associated vasculopathy with onset in infancy (SAVI), manifest clinical and laboratory features akin to SLE (Table 1). These conditions involve dysregulated IFN-I signaling, leading to a cascade of immune dysregulation and autoimmune-like phenotypes resembling SLE, such as the presence of autoantibodies, skin manifestations, and neurological abnormalities. Therefore, while monogenic SLE typically results from mutations in genes directly linked to lupus susceptibility, there is a notable convergence between certain type I interferonopathies and SLE, emphasizing the pivotal role of IFN-I dysregulation in the pathogenesis of both monogenic and multifactorial forms of SLE. Understanding these overlaps provides insights into shared immunopathogenic mechanisms and highlights the intricate interplay between genetic factors and aberrant interferon signaling in autoimmune diseases.

**Table 1:** Genes related to type I interferonopathies with systemic lupus erythematosus phenotype

Related processes	Disease-causing genes
Nucleic acid metabolism	*TREX1* (LOF, AR or DN), *RNASEH2B* (LOF, AR), *RNASEH2C* (LOF, AR), *RNASEH2A* (LOF, AR), *SAMHD1* (LOF, AR), *ADAR1* (LOF, AR or DN), *DNASE1* (LOF), *DNASE2* (LOF, AR), *DNASE1L3* (LOF, AR), *LSM11* (LOF, AR), *RNU7-1* (LOF, AR)
Nucleic acid sensing	*IFIH1* (GOF, AD), *DDX58* (GOF, AD), *TLR8* (GOF, XL)
Nucleic acid signaling	*STING1* (GOF, AD/AR), *COPA* (LOF, DN), *ARF1* (LOF, DN), *OTUD1* (LOF)
Post interferon receptor signaling	*JAK1* (GOF, AD), *USP18* (LOF, AR), *ISG15* (LOF, AR), *STAT2* (LOF, AR), *SOCS1* (haploinsufficiency, AD), *STAT1* (GOF, AD)
Proteasome	*PSMB4* (LOF, AR), *PSMB8* (LOF, AR), *PSMB9* (LOF, AR), *PSMB10* (LOF, AR), *PSMA3* (LOF, AR), *PSMA5* (uncertain), *PSMC5* (LOF, DN), *PSMG2* (LOF, AR), *PSMD12* (haploinsufficiency, AD)
Unclear	*ACP5* (LOF, AR), *RELA* (LOF, DN), *SAMD9L* (AD)

*LOF* loss-of-function, *AD* autosomal dominant, *DN* dominant negative, *AR* autosomal recessive, *GOF* gain of function, *XL* X-linked, *TREX1* 3′ repair exonuclease 1, *RNASEH2A* ribonuclease H2 subunit A, *SAMHD1* SAM-domain- and HD-domain-containing protein 1, *ADAR1* adenosine deaminase RNA specific 1, *DNASE1L3* deoxyribonuclease 1 like 3, *IFIH1* interferon induced with helicase C domain 1, *TLR* Toll-like receptor, *STING1* stimulator of interferon response cGAMP interactor 1, *COPA* coatomer protein complex subunit alpha, *ARF1* ADP-ribosylation factor 1, *OTUD1* ovarian tumor deubiquitinase 1, *JAK1* Janus kinase 1, *USP18* ubiquitin-specific peptidase 18, *ISG15* interferon-stimulated gene 15, *STAT* signal transducer and activator of transcription, *SOCS1* suppressor of cytokine signaling 1, *PSM* proteasome, *SAMD9L* sterile alpha motif domain containing 9 like

### Aicardi–Goutières syndrome

AGS was first reported in 1984 by neurologists Jean Aicardi and Françoise Goutières. To date, seven related pathogenic genes have been discovered, including 3′ repair exonuclease 1 gene (*TREX1*; AGS1), ribonuclease H2 subunit B gene (*RNASEH2B*; AGS2), *RNASEH2C* (ASG3), *RNASEH2A* (AGS4), SAM-domain- and HD-domain-containing protein 1 gene (*SAMHD1*; AGS5), adenosine deaminase RNA specific 1 gene (*ADAR1*; AGS6), and interferon induced with helicase C domain 1 gene (*IFIH1*; AGS7) [32]. TREX1, also known as DNase III, is a 3′-5′ DNA exonuclease present in the cytoplasm, and it acts on single-stranded DNA (ssDNA) and double-stranded DNA (dsDNA). TREX1 cleaves mismatched and modified nucleotides from the 3′ end of DNA, degrading DNA from retroviruses and reverse transcriptase [33]. SAMHD1 degrades deoxynucleoside triphosphates (dNTPs) into 2′-deoxynucleosides and triphosphate subunits, thereby stably consuming the cellular dNTP pool and preventing the accumulation of cytoplasmic ssDNA [34]. RNASEH2, ribonuclease H2, is an enzyme composed of three subunits, including the catalytic subunit RNASEH2A and the non-catalytic subunits RNASEH2B and RNASEH2C. RNASEH2 degrades the RNA portion of RNA/DNA hybrids and hydrolyzes the phosphodiester bonds of ribonucleotides embedded in DNA double strands, preventing genomic instability and the accumulation of abnormal nucleic acids [35]. Their deficiencies lead to the accumulation of ssDNA, dsDNA, and RNA/DNA hybrids in the body, which are recognized by cGAS. This triggers the activation of STING, which in turn recruits TBK1 to phosphorylate IRF3, leading to the production of IFN-β.

ADAR1 primarily acts on RNA as an adenosine deaminase, catalyzing the hydrolytic deamination of adenosine to inosine in double-stranded RNA (dsRNA) [36]. Loss of ADAR1 function leads to an increase in dsRNA levels. MDA5, also known as IFIH1, recognizes dsRNA, inducing the activation and oligomerization of mitochondrial antiviral signaling proteins [37]. This activates TBK1 and IκB kinase, ultimately resulting in the activation of the transcription factors nuclear factor (NF)-κB, IRF3, and IRF7. These transcription factors translocate to the cell nucleus, where they promote the production of IFN-I.

Initially, AGS was thought to manifest as progressively worsening neurological symptoms, including spasms, hypertonia, and microcephaly, with severe cases resulting in early death. Patients with AGS exhibit elevated levels of IFN-stimulated genes (ISGs) and IFN-α in their blood and cerebrospinal fluid. As more cases have been documented, a wider range of clinical phenotypes have emerged, including chilblain-like skin rashes, intermittent fever, cytopenia, hepatosplenomegaly, elevated transaminases, hypothyroidism, interstitial lung disease, intracranial calcifications, and white matter lesions in the brain, accompanied by positive autoantibodies, leading to a misdiagnosis of SLE [35].

### *DDX58* gain-of-function

*DDX58* encodes retinoic acid-inducible I (RIG-I), an intracytoplasmic nucleic acid sensor. The hyperactivation of RIG-I impairs ATPase activity, leading to constitutive self-RNA recognition and IFN-I pathway activation [38]. Initially, *DDX58* gain-of-function was thought to present as atypical Singleton–Merten syndrome, characterized by abnormal valvular and thoracic calcifications and osteoporosis [39]. Recently, a new *DDX58* pathogenic variant, R109C, was found to be associated with lupus nephritis [40]. These patients manifested autoimmune symptoms, including nephritis, hemolytic anemia, positive antinuclear antibodies (ANAs), anti-dsDNA and anticardiolipin antibodies and decreased C3, accompanied by an elevated IFN signature. Further investigations indicated that compared with previous variants, the R109C variant leads to a loss of RIG-I autoinhibition, which is a distinct pattern.

### Abnormalities in toll-like receptors

TLRs are single-spanning membrane proteins located on the surface or within endosomes of immune cells. Defects in TLR signaling pathways are linked to increased susceptibility to bacterial infections and compromised inflammatory responses during infections. Conversely, gain-of-function mutations in both *TLR7* and *TLR8*, which detect both viral and bacterial single-stranded RNA [41], are related to SLE [42, 43]. TLR8 is found in monocytes/macrophages, myeloid dendritic cells, and granulocytes, while TLR7 is present in plasmacytoid dendritic cells, B cells, and monocytes/macrophages [44]. Upon stimulation, these receptors activate NF-κB, mitogen-activated protein kinase (MAPK), and IRF, leading to the transcription of inflammatory cytokines, chemokines, and costimulatory molecules. However, their downstream signaling pathways differ across cell types. In monocytes, TLR8 activation triggers robust NF-κB and IFN-I responses, promoting T helper (Th) 1 cytokine production. Conversely, TLR7 primarily activates MAPK signaling and induces Th17 cytokines. Notably, TLR7 inhibits the TLR8-mediated IFN-I response [45]. With respect to *TLR8* gain-of-function, patients often exhibit early-onset severe cytopenia, hepatosplenomegaly, and lymphadenopathy accompanied by progressive autoinflammatory features, including fever, arthritis and central nervous system vasculitis. Laboratory examinations revealed elevated proinflammatory cytokines, including interleukin (IL)-18, IL-1β, and tumor necrosis factor (TNF)-α, as well as an increased IFN signature [42, 46], indicating the possibility of interferonopathies. However, this is still unclear since the IFN signature associated with *TLR8* gain-of-function is lower than that associated with classical type I interferonopathies [46]. In addition, *TLR7* gain-of-function did not increase the IFN signature [43].

### Deoxyribonuclease deficiencies

Deoxyribonucleases (DNases) are a group of enzymes responsible for catalyzing the degradation of DNA molecules, thus preventing the recognition of self-DNA. DNase I is a prominent serum endonuclease primarily responsible for degrading extracellular dsDNA from dying cells. DNase II is a crucial lysosomal endonuclease that plays a pivotal role in breaking down exogenous DNA encountered through endocytosis. Deoxyribonuclease 1 like 3 (DNase1L3) shares homology with DNase I and is presumed to play a role in the clearance of NETs. Deficiencies in these enzymes cause the accumulation of nucleic acid, leading to the activation of DNA sensors and the IFN-I signaling pathway [47].

In terms of DNase I deficiency, mice deficient in *DNASE1* develop ANA and glomerulonephritis [48]. There have been reports of several cases of SLE in which individuals had a mutation in *DNASE1*, resulting in markedly elevated levels of anti-nucleosome antibodies [49, 50].

Clinical manifestations of DNase II deficiency are similar to those of AGS and may present with symptoms in the neonatal period, including hepatosplenomegaly, cholestatic hepatitis, and, in severe cases, progression to cirrhosis. Additionally, patients may exhibit chilblain-like skin rashes, cytopenia, proteinuria, non-destructive deformative joint disease, neurological symptoms, and intracranial calcifications, and some may show features of immunodeficiency and autoimmune diseases [51].

The absence of DNASE1L3 can lead to early-onset and clinically severe SLE. In 2011, Al-Mayouf et al. reported 17 patients with SLE from six unrelated families attributed to DNASE1L3 deficiency. All of these patients developed childhood-onset SLE, with ages of onset ranging from 2 to 12 years. Notably, all patients tested positive for ANA and ds-DNA antibodies but also displayed decreased C3 and C4 levels. Among this group, 11 patients were positive for anti-neutrophil cytoplasmic antibodies (ANCAs), and 11 were diagnosed with lupus nephritis [52].

### LSM11 deficiency and RNU7-1 deficiency

LSM11 and RNU7-1 are two components of the replication-dependent histone pre-mRNA processing complex. In 2020, Uggenti et al. discovered that biallelic mutations in either *LSM11* or *RNU7-1* led to a dysregulation of histone messenger RNA transcripts, as well as a disturbance of histone protein composition, which further activated interferon signaling through a cGAS–STING-dependent pathway [53]. According to previous reports, patients exhibit typical AGS phenotypes.

### STING-associated vasculopathy with onset in infancy

STING is a transmembrane protein encoded by *STING1* that is located primarily in the endoplasmic reticulum and serves as an essential sensor of self-DNA molecules. Generally, STING is activated by its ligand cGAS, triggering the activation of the IFN-I signaling pathway via TBK1 and IRF3. However, gain-of-function mutations in *STING1* can result in ligand-independent activation of STING, leading to sustained activation of the IFN-I signaling pathway [54]. SAVI is characterized by interstitial lung disease, cutaneous involvement such as erythematous or purpuric plaques and nodules, livedo reticularis, and painful ulcerative lesions, as well as systemic symptoms such as failure to thrive, fever, malaise, and chronic anemia, sometimes accompanied by positive autoantibodies such as low-titer ANA or positive anti-phospholipid antibodies [55, 56].

### COPA syndrome

Coatomer protein complex subunit alpha (COPA) syndrome is caused by a heterozygous mutation in the *COPA* gene, which encodes the α-subunit of coat protein complex I (COPI) and is involved in the trafficking of membranes from the Golgi apparatus to the endoplasmic reticulum [57]. Defects in the COPI complex lead to prolonged activation of STING in the Golgi apparatus, resulting in the overexpression of IFN-I. The clinical characteristic of COPA syndrome is lung disease (including cystic, hemorrhagic, and interstitial lung disease), with a smaller subset also experiencing arthritis and kidney disease. Laboratory studies may reveal ANA and ANCAs. In a recent report, a 10-year-old girl who was diagnosed with lupus nephritis and carried a mutation in the *COPA* gene indicated that COPA syndrome could also be a cause of lupus nephritis [58]. Another study suggested that alveolar hemorrhage and pulmonary vasculitis in both SLE patients and COPA syndrome patients might be initiated by endothelial injury, resulting in endoplasmic reticulum stress, lung endothelial cell apoptosis and myeloid cell recruitment [59].

### ARF1 deficiency

ADP-ribosylation factor 1 (ARF1) is a negative regulator of cGAS-STING signaling. It was recently found to be related to interferonopathy. Heterozygous *ARF1* missense mutations produce GTPase-defective ARF1, which leads to prominent mitochondrial DNA release and the accumulation of STING, resulting in increased IFN-I signaling [60]. All patients exhibited significant developmental delay, and 75% (three of four) of patients had skin lesions such as chilblain lupus. Notably, no patients had epilepsy, and images of the central nervous system were normal in all patients, without intracranial calcification or other manifestations observed in AGS patients.

### OTUD1 deficiency

The ovarian tumor deubiquitinase 1 gene (*OTUD1*) encodes a deubiquitinase enzyme that plays a pivotal role in cellular processes by interacting with IRF3. Its primary function involves the deubiquitination of IRF3, leading to the removal of polyubiquitin chains from this transcription factor and subsequently resulting in the suppression of *IFN* gene transcription. However, when loss-of-function (LOF) missense mutations occur within the *OTUD1* gene, this finely tuned regulatory mechanism is disrupted. Consequently, these mutations cause the overactivation of *IRF3*, leading to the aberrant upregulation of *IFN* genes. This dysregulation of the immune response has been closely associated with the development of a diverse array of autoimmune diseases, which notably include early-onset SLE [61].

### *JAK1* gain-of-function

Janus kinase 1 (JAK1) is one of the most important kinases downstream of IFN-I, and the *JAK1* gain-of-function variant A634D was reported to cause a syndrome characterized by hepatosplenomegaly, eosinophilia, enteritis, thyroid disease, growth retardation, and susceptibility to viral infection [65, 66]. Membranous nephropathy has also been reported [66]. Tofacitinib, a pan-JAK inhibitor, has been reported to ameliorate clinical and biological immune dysfunction [66].

### USP18 deficiency and ISG15 deficiency

Ubiquitin-specific peptidase 18 (USP18) downregulates IFN-I signaling by inhibiting the interaction between JAK1 and IFN-α receptors (IFNARs), while interferon-stimulated gene 15 (ISG15) is a ubiquitin-like protein induced by IFN-I that can bind to USP18, thereby protecting USP18 from proteasome degradation and enhancing its suppressive function. Defects in either ISG15 or USP18 can ultimately cause sustained activation of the IFN-I signaling pathway [67, 68]. Patients typically exhibit a broad range of neurological and immunological manifestations. These can include basal ganglia calcification, seizures, and susceptibility to mycobacterial infections. Additionally, these patients often display higher levels of autoantibodies than healthy individuals [69].

### STAT2 deficiency

In 2019 and 2020, two reports suggested that homozygous mutations, specifically the p.Arg148Trp or p.Arg148Gln separation-of-function mutations in the *STAT2*, resulted in the inability of the protein to interact with USP18. This interaction is pivotal for the recruitment of USP18 to IFNAR2. As a consequence of these mutations, patients exhibit severe neuroinflammatory diseases characterized by progressive intracranial calcification, white matter disease, and intracranial hemorrhage. In addition to these neurological symptoms, patients also exhibit systemic inflammation and multisystemic dysfunction, which includes recurrent fever, hepatosplenomegaly, cytopenia with pronounced thrombocytopenia, elevated ferritin levels, heightened liver enzyme activity, and nephrotic range proteinuria [70, 71].

### *SOCS1* haploinsufficiency

Suppressor of cytokine signaling 1 (SOCS1) haploinsufficiency was initially reported in 2020, and it is primarily attributed to monoallelic LOF variants (large deletions, frameshift mutations, and nonsense variants) [72–75]. SOCS1 regulates the JAK-STAT pathway by inhibiting JAK1/2 phosphorylation and suppressing the activity of TYK2 [76]. Thus, individuals with SOCS1 dysfunction exhibit an enhanced IFN signature. Based on limited published reports to date, disease onset typically occurs within the first decade of life. Patients may present with various rheumatologic manifestations, including polyarthritis, recurrent mucosal ulcerations, fever, glomerulonephritis, alopecia, autoimmune endocrinopathies, psoriasis, and features resembling SLE. There are also other manifestations, including gastrointestinal tract abnormalities (hepatosplenomegaly, hepatitis, and colitis), recurrent infections, variable T-cell lymphopenia, and natural killer cell dysfunction. Furthermore, secondary granulomatous interstitial lung disease related to immune dysregulation has been documented. Notably, recent findings have revealed benign variants of *SOCS1* in cis, leading to early-onset SLE-like symptoms. This finding underscores the significance of SOCS1 in cytokine signaling, as even a partial disruption of one allele by approximately 20%–30% may suffice to drive aberrant IFN-I signaling [77].

### *STAT1* gain-of-function

Individuals with *STAT1* gain-of-function mutations exhibit increased expression of ISGs in whole blood. However, it is essential to note that *STAT1* mutations involve the activation of multiple cytokine pathways, in addition to the IFN-I signaling pathway. This multifaceted cytokine activation likely contributes to aspects of their clinical phenotypes that are not typically observed in other type I interferonopathies. While some patients with *STAT1* gain-of-function mutations present with intracranial calcification and aortic calcification, the core clinical phenotype primarily includes chronic mucocutaneous candidiasis and autoimmune hypothyroidism [78–81].

### Proteasome-associated autoinflammatory syndrome

Dysfunction of immune proteasomes, whose primary role is the hydrolysis of intracellular senescent and exogenous proteins, is characterized by this process. This dysfunction is attributed to mutations in immunoproteasome subunits and constituent subunits [proteasome subunit alpha type 3 (*PSMA3*)*, PSMA5, PSMB4, PSMB8, PSMB9, PSMB10*], regulatory particles (*PSMC5, PSMD12*), and proteasome assembly units [*POMP* (proteasome maturation protein)*, PSMG2*]. These mutations result in diminished enzymatic hydrolysis activity and contribute to proteasome-related diseases [82–86]. Notably, in 2023, Papendorf et al. reported the discovery of a novel variant in *PSMC5*, a gene previously unassociated with the proteasome. However, it is essential to note that this finding cannot be definitively attributed to the condition, as the patient also possessed a pathogenic maternally inherited *PSMB8* variant and a de novo *PSMA5* mutation [86]. Common clinical features of this disorder include the presence of pernio-like purplish nodular lesions (neutrophilic dermatosis), panniculitis accompanied by progressive lipodystrophy and muscle atrophy, and joint contractures leading to extremity deformities. Hepatosplenomegaly and hypochromic or hemolytic anemia have also been reported. Early metabolic syndrome, characterized by systemic hypertension and dyslipidemia, affects 40%–80% of patients [84, 87–89]. Furthermore, proteasome defects have been associated with neurological diseases, such as the development of microcephaly and cognitive delay [90]. Clinical studies have noted elevated levels of IFN-I in PRAAS patients. Nevertheless, the precise molecular mechanisms underlying the enhanced IFN levels and their connection to proteasome dysfunction remain to be elucidated.

### Spondyloenchondrodysplasia with immune dysregulation

The clinical manifestations of spondyloenchondrodysplasia with immune dysregulation (SPENCDI) associated with *ACP5* mutations are similar to those of AGS in terms of neurological manifestations (spasticity, intracranial calcifications) as well as manifestations of autoimmunity (mimicking SLE) [62, 63]. Autosomal recessive mutations in the *ACP5* gene lead to a deficiency of tartrate-resistant acid phosphatase (TRAP), which is normally expressed in osteoclasts and myeloid cells. TRAP plays a crucial role in the processing and degradation of osteopontin (OPN) within plasmacytoid dendritic cells by catalyzing the dephosphorylation of OPN. Defects in TRAP cause prolonged phosphorylation of OPN, which leads to sustained activation of the TLR9 pathway, inducing the nuclear translocation of the *IRF7* and NF-κB transcription factors and ultimately resulting in increased expression of IFN-I, IL-6, and TNF [64].

### *RELA* dominant-negative mutation

*RELA* encodes the p65 protein, a member of the NF-κB family of transcription factors. Earlier research indicated that *RELA* haploinsufficiency leads to chronic mucocutaneous ulceration and autoimmune hematological disorders, largely dependent on TNF [91]. However, a recent study showed that patients with *RELA* dominant-negative (DN) mutations exhibit clinical features similar to those of patients with *RELA* haploinsufficiency plus inflammatory symptoms such as periodic fever, inflammatory bowel diseases, juvenile idiopathic arthritis, and skin involvement such as erythema nodosum or pustulosis [92]. Basic research has shown that myeloid cells with *RELA* DN mutations have increased TLR7 and myeloid differentiation factor 88 expression and thus produce more IFN-I in response to TLR7 activation [92].

### SAMD9L-associated autoinflammatory disease

It is caused by truncating mutations within the nucleotide-binding oligomerization domain of *SAMD9L*, which plays a role as an autonomous brake to block the translation of several proteins to inhibit growth factor signaling [93] and cell cycle progression and stimulate inflammation [94]. The truncating mutations resulted in the absence of the winged helix domain/helical domain 2 and tetracopeptide repeat domain, obscuring the nucleotide-binding domain oligomerization interface. Consequently, the truncated protein tends to multimerize into its active form, inhibiting mRNA translation even in the absence of viral RNA triggering [95]. These patients often exhibit early-onset multisystemic inflammation, including panniculitis (characterized by neutrophil inflammation), interstitial pneumonia, intracranial calcification, pancytopenia, and highly elevated inflammatory markers (C-reactive protein and erythrocyte sedimentation rate) [94]. These immunophenotypes suggested a progressive decrease in B cells and natural killer cells.

**Fig. 2:**
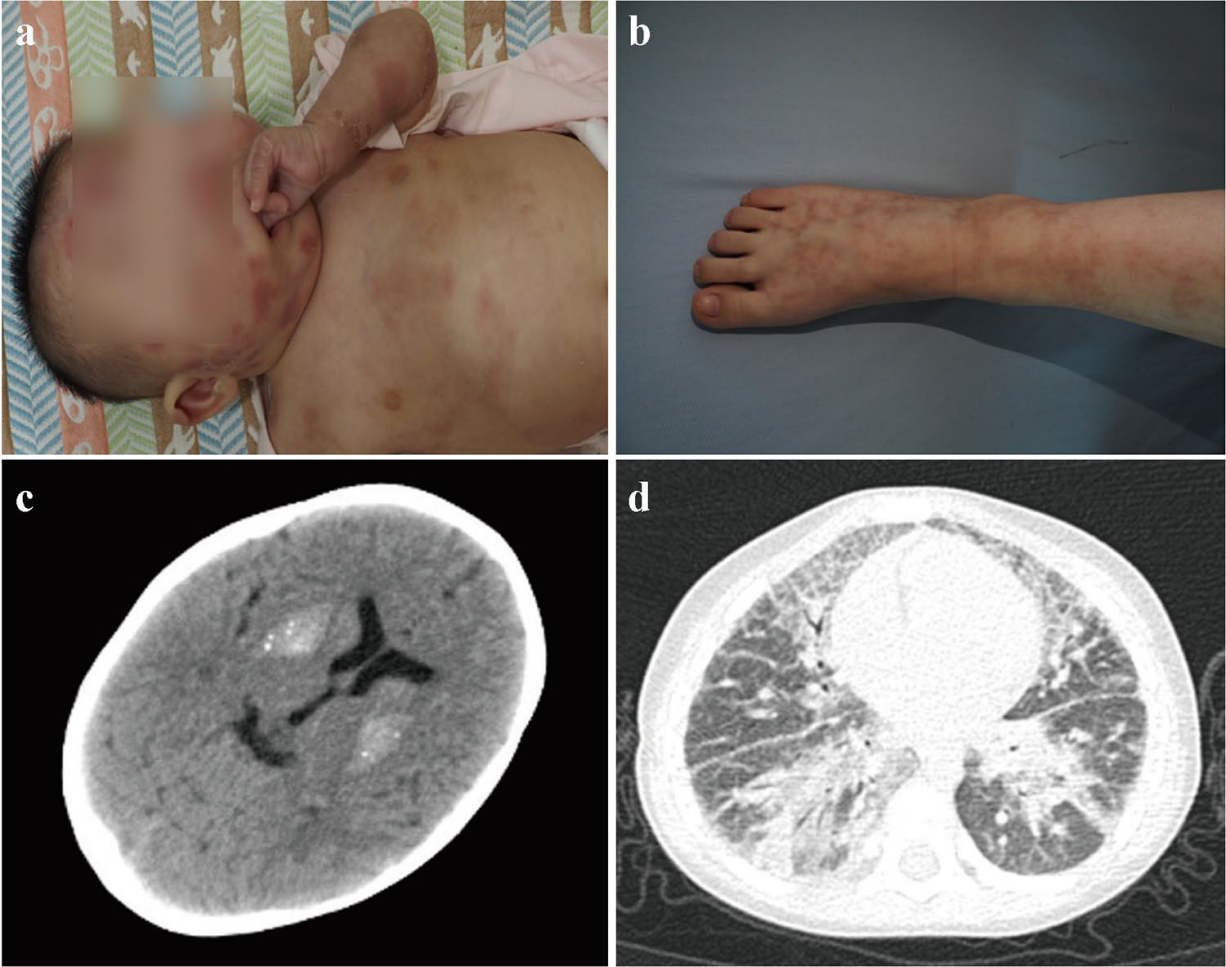
Images showing typical manifestations of type I interferonopathies (permissions were obtained by parents for publication). **a** Chilblains; **b** livedo reticularis; **c** intracranial calcification; **d** interstitial lung disease

All interferonopathies discussed above could present as SLE-like symptoms, and thus, it is important to identify them from among all patients with suspected SLE. Importantly, compared to patients with classical SLE, patients with type I interferonopathies usually exhibit several specific clinical manifestations, including early onset, skin vasculopathy (chilblains, livedo reticularis and panniculitis, as shown in Fig. 2), central nervous system involvement (intracranial calcification, as shown in Fig. 2; seizures and psychomotor development delay), interstitial lung disease (as shown in Fig. 2), elevated transaminases and hypothyroidism. In addition, since patients with these interferonopathies could present with SLE, the study of type I interferonopathies could further reveal the pathogenesis of SLE and provide potential therapeutic targets in the future.

## Measurement of the activity of IFN-I signaling in SLE

Efforts have been made to establish assays for detecting biomarkers associated with the IFN-I pathway since they have the potential to diagnose and stratify patients. In 2023, Burska et al. conducted a literature review including 276 papers on IFN-I assays, and those assays included immunoassays for IFN-I proteins (*n* = 58), single-molecule assays (SimoA; majorly measuring IFN-α, *n* = 8), immunoassays for IFN-inducible proteins (*n* = 42), flowcytometry for IFN-I-inducible markers (*n* = 12), RNA microarrays for IFN signatures, scores or modules of ISGs (*n* = 70), RNA sequencing (*n* = 9), NanoString for the expression of ISGs (*n* = 5), IFN-I scoring by quantitative polymerase chain reaction (*n* = 122) and others [96].

The widely used assay for assessing interferon pathway activation is to analyze the expression of ISGs. Usually, a set of the most strongly and consistently expressed ISGs is selected, and one of the commonly used sets consists of six genes: *IFI27*, *IFI44L*, *IFIT1, ISG15*, *RASD2*, and *SIGLEC1* [97]. It is the most developed technique, and many centers have used it routinely [98]. Nevertheless, studies indicate that different clinical conditions may have distinct subsets of ISGs expressed, which means that a given subset of ISGs might not be sensitive for detecting activation [99]. In addition, there is still some overlap between the IFN-I and IFN-II pathways, and thus, an increase in the ISG could sometimes be a consequence of IFN-II pathway activation instead of IFN-I [100–102].

In early studies, detecting IFN-α, whose half-life is 2–3 hours [103], was difficult due to its relatively low concentration in serum [104]. With the help of sensitive SimoA methods, several studies have attempted to measure the concentrations of IFN-I [105]. A study consisting of 48 pediatric SLE patients and 67 healthy controls demonstrated that serum IFN-α2 levels are strongly positively correlated with the IFN-I gene signature and that serum IFN-α2 levels are significantly associated with both the safety of estrogens according to the Lupus Erythematosus National Assessment-SLE disease activity index (SLEDAI) and the British Isles Lupus Assessment Group 2004, while the IFN-I gene signature did not show this association [106]. Nevertheless, soluble IFN-α2 is only a member of the IFN-I family and might not be able to represent the total activity of IFN-I. In addition, this assay is only able to measure the level of IFN-α2 in serum and is not sufficient to reflect specific local conditions, such as those in the kidney or joints.

There are many other attempts to find more assays that are convenient for detecting IFN-I activation in clinical practice. The detection of surface molecules by flow cytometry seems to be promising. CD169, also known as Siglec-1, is specifically expressed on monocytes, dendritic cells and tissue macrophages. It is induced by stimulation with IFN-I, including IFN-α, IFN-β and IFN-ω, but not IFN-γ. Sakumura et al. demonstrated that CD169 expression on CD14^+^ monocytes, as detected by flow cytometry, is elevated in pediatric SLE patients and parallels the concentration of IFN-α [107]. Additionally, Hoogen’s group showed that Galectin-9 is correlated with disease activity and serves as a reliable and easily measurable serum biomarker for detecting the interferon signature in patients with SLE [108]. Their conclusion is further supported by Yuksel’s study, which showed that the serum level of Galectin-9 is correlated with the SLEDAI [109]. The expression of *SAMHD1* was also suggested to be associated with the IFN-I signaling pathway. By examining 98 pediatric SLE patients and 44 gender- and age-matched healthy donors, *SAMHD1* was found to be positively correlated with several ISG, including myxovirus resistance protein A, *IRF3* and *IRF7* [110].

As concluded by the 2023 European Alliance of Associations for Rheumatology points to consider, current measurement assays measure various aspects of the IFN pathway, but they do not provide a comprehensive assessment of the entire pathway, and some lack specificity for IFN-I. The selection of the most appropriate assay depends on the specific research or clinical question [111].

## Clinical implications of IFN-I in SLE

Since the IFN-I pathway plays an essential role in SLE pathogenesis, the detection of IFN-I is widely used in the diagnosis, stratification, and prognosis of patients with SLE. In addition, anti-IFN treatments are assumed to be a potential method for treating some specific SLE patients. In terms of the application of IFN-I in the diagnosis of SLE. In 2018, Wahadat et al. reported that 57% of pediatric SLE patients had a positive IFN-I signature [112]. Another study performed by Zorn-Pauly et al. suggested that in newly diagnosed SLE patients, almost all patients had IFN-I pathway activation, and a negative flow cytometry result for CD169 is efficient for excluding SLE, whose negative predictive value is > 99% [113].

Assays testing IFN-I pathway activation also have an important role in the stratification of patients with SLE. By using blood genomics, researchers identified three subtypes of SLE: “IFN-high”, “NE-high” and “mixed” [114]. They assumed that high levels of interferons (IFN-high) may arise due to the excessive production of IFNs triggered by viral infections, subsequently leading to the development of autoantibodies. On the other hand, elevated levels of NETs (NE-high) may predominantly result from infections caused by bacteria and fungi, which stimulate neutrophils to generate NETs and induce individual autoimmune responses. They also showed that the “mixed” group had the highest SLEDAI scores, while the “NE-high” group tended to cluster with healthy patients. Another group discovered a distinct clinical subset of pediatric SLE patients using interferon scores and complement levels [115]. These studies suggested that these patients had lower anti-dsDNA levels and might represent a predominant autoinflammatory subset of pediatric SLE patients. However, in their cohort, the IFN score was not correlated with disease activity, which contradicts the results of other groups. Several attempts have been made to identify differences between SLE patients with a high IFN signature and those with a low IFN signature, but the results were not satisfactory. There was a study suggesting significant associations between a high IFN signature and higher titers of anti-dsDNA, elevated serum B-cell activating factor and hypocomplementemia but no correlations with clinical disease activity [116].

Additionally, some markers of IFN-I are related to specific phenotypes. It was suggested that IFN-α and neopterin in cerebrospinal fluid (CSF) constitute promising biomarkers of neuropsychiatric SLE in a 5-year retrospective monocentric pediatric SLE cohort [117]. CSF IFN-α levels are significantly greater in patients with active neuropsychiatric SLE than in patients with inactive SLE, while the serum IFN-α levels are similar. In addition, correlating with the improvement in symptoms of neuropsychiatric SLE, the CSF concentration of IFN-α decreased. SAMDH1, as mentioned above, was also indicated to be related to vasculitis. Its levels were significantly increased (*P* < 0.05) in pediatric SLE patients with butterfly erythema, alopecia, and photosensitivity [110].

The prominent role of IFN-I in SLE pathogenesis has also spurred the development of targeted therapies. Anti-IFN therapies, including sifalimumab (an anti-IFN-α monoclonal antibody) and anifrolumab (a monoclonal antibody targeting the IFN-I receptor), have demonstrated favorable outcomes in phase II randomized controlled trials involving adult SLE patients with moderate-to-severe disease [118–120]. These therapies, when used in conjunction with standard-of-care medications, have shown efficacy in the absence of severe nephritis and neuropsychiatric involvement. However, pediatric patients were not included in those trials, and thus, the efficacy and efficacy of these biologics in pediatric populations still need further investigation. Additionally, JAK inhibitor treatment is an alternative therapeutic approach for pediatric patients with severe pediatric SLE. It has shown promise in gradually reducing inflammatory markers and IFN scores and promoting the upregulation of the DNA repair pathway [121]. Many molecules, such as inhibitors of TBK1 and IRF5, are also able to partially inhibit the IFN pathway, showing promising results in in vitro experiments [112, 122].

## Conclusions

The role of IFN-I in pediatric-onset SLE is complex and multifaceted, as highlighted in this comprehensive review. While much of the existing research on IFN-I has focused on adult SLE patients, it has become increasingly evident that IFN-I signaling plays a pivotal role in the pathogenesis of pediatric SLE. Several key insights and implications emerge from this review: (1) IFN-I signaling in pediatric SLE: robust evidence demonstrating that IFN-I signaling is significantly activated in pediatric SLE patients; (2) genetic variants and susceptibility: genetic factors, such as variants in IFN-I-related genes such as *IRF5* and *TYK2*, have been identified as risk factors for pediatric SLE. These genetic associations shed light on the genetic underpinnings of the disease and provide potential targets for future research and therapeutic interventions; (3) type I interferonopathies: this review highlights the overlap between type I interferonopathies and pediatric SLE. These conditions, characterized by sustained IFN-I activation, can closely mimic SLE symptoms, underscoring the complex interplay between genetic factors and immune dysregulation in autoimmune diseases. It is important to be aware of the possibility of type I interferonopathies in patients with SLE. On the other hand, the study of type I interferonopathies provides a way to discover the pathogenesis of SLE and potential therapeutic targets in the future; and (4) diagnostic and therapeutic implications: the detection of IFN-I activation has significant diagnostic and therapeutic implications for pediatric SLE patients. Biomarkers such as ISG signatures, CD169 expression, and Galectin-9 levels hold promise for improved diagnosis, stratification, and monitoring of disease activity in pediatric patients. Additionally, the emergence of anti-IFN therapies and JAK inhibitors presents potential treatment options, although their efficacy and safety in pediatric populations require further investigation.

In conclusion, this review underscores the pivotal role of IFN-I signaling in the pathogenesis of pediatric SLE and its potential as a diagnostic marker and therapeutic target. Additionally, this review describes the manifestations of interferonopathies with monogenic SLE presentations as well as the pathogenic mechanism of related genes, highlighting the importance of identifying those diseases, especially for patients with early-onset clinical IFN signatures, skin vasculopathy with chilblains, livedo reticularis and panniculitis, involvement of the central nervous system, and interstitial lung disease. Further research, particularly in the pediatric population, is needed to refine our understanding of the precise contributions of IFN-I to disease progression and to translate this knowledge into more effective and personalized management strategies for pediatric SLE patients. The complex interplay between genetic, immunological, and environmental factors in pediatric SLE patients requires continued investigation to improve patient outcomes and quality of life.

## Data Availability

All data generated or analysed during this study are included in this published article.
